# Investigation of L2/Ln Pragmatic Competence: Its Core and Route Map

**DOI:** 10.3389/fpsyg.2021.690550

**Published:** 2021-08-17

**Authors:** Tiaoyuan Mao

**Affiliations:** Department of Language and Literature, Soochow University, Jiangsu, China

**Keywords:** pragmatic competence, core properties, outline, thought, pathological exploration

## Abstract

How to use language properly and acquire the capacity for language use has become the focus of linguists and philosophers for centuries. Therefore, pragmatic competence underlying language use arouses enormous interests of language acquisition practitioners. This study reveals the core properties of various models or theories of pragmatic competence, such as the communicative componential models, the form-function mapping proposal of the functionalist, the tripartite cognitive model, and the current integrated model of pragmatic competence. The common core includes (but not limited to) integration of thought and communication, one uniform pragmatic mechanism, dynamic form-function mapping, and complementarity between grammatical and pragmatic competences. With the findings as a departure, a brief outline for further investigation of pragmatic competence is proposed finally, including pathological and neurobiological examination of pragmatic competence.

## Introduction

Pragmatic competence is an essential construct in language development in both children and adults. Normally, it refers to the capacity to use language effectively in a context (Thomas, 1983). Along this line, the investigation of L2 pragmatic competence almost concentrates on the sociocultural development of pragmatic competence (for a review, see Timpe et al., 2015; Culpeper et al., 2018), so do the assessment of L2 pragmatic competence (Liu, 2006; Bachman and Palmer, 2010; Bardovi-Harlig and Shin, 2014 a.o.) and the teaching of L2 pragmatic competence (cf. Rose and Kasper, 2001; Taguchi, 2010, 2019). The reason why the exploration of L2 pragmatic competence is narrowed down to the particular sociocultural aspect might attribute to the unclear profile of pragmatic competence *per se*. From the first conceptualization of pragmatic competence by Chomsky (1977) to the well-known clarification of its communicative purpose in applied linguistics (cf. Thomas, 1983), its definition is not unanimously accepted, and its specific operative mechanism is not clearly expounded either. Even though some proposals are formulated to clarify the operation of pragmatic competence, there are still some debates about whether the existing operative mechanisms could realize the true nature and function of pragmatic competence (cf. Timpe et al., 2015; Mao and Dai, 2017; Mao and He, 2021). Basically, what is commonly known about pragmatic competence is almost the relevant sociocultural norms or knowledge that are involved in its development and assessment.

Thanks to the innovation in theoretical linguistic theories, especially, the resolute improvement in the current Minimalist Program of bio-linguistic paradigm, researchers can intensively probe into the nature of pragmatic competence and formulate a new model of pragmatic competence. Such being the case, a comparison between the new model under the bio-linguistic paradigm and other influential proposals of pragmatic competence from various linguistic perspectives will definitely reveal more about the core properties of pragmatic competence and its operative mechanism. Furthermore, being familiar with the core properties and mechanism of pragmatic competence will positively deepen our understanding of the nature of language use, ushering in a new tendency in the investigation of the social and theoretical values of pragmatic competence, such as the pathological diagnosis of autism, and the possibility to improve the current linguistic theories and clarify some complicated issues in philosophy of language. As a result, it is worth expounding to what extent the core properties revealed in the comparison will initiate a new research trend.

This study consists of four sections, with “Introduction” as its first section. The second section “The core of pragmatic competence” examines existing proposals and a newly figured one, namely, the integrated model of pragmatic competence (IMPC) (Mao, 2020; Mao and He, 2021), and investigates the shared features of all models or proposals of pragmatic competence. The third section “Route map for further exploration of L2/Ln pragmatic competence” presents the outline for the current and featural exploration of L2 or Ln pragmatic competence on the basis of what we know. The fourth section “Conclusion” concludes the study.

## The Core of Pragmatic Competence

As is known, the critique of “the classic dichotomy of competence and performance” (Chomsky, 1965/2015) by sociolinguists ignites the subsequent creation of “the communicative competence” (Hymes, 1972) and the separation of grammatical and pragmatic competences (Chomsky, 1977). Most applied linguists follow Hymes' suit, exploring to what extent sociocultural factors facilitate the mastering language ability and language use of the common people. Thus, several proposals of pragmatic competence, serving various academic purposes such as language assessment and teaching, have been built up. While in generative linguistics, the proposal is under investigation, and there is no explicitly clarified proposal for pragmatic competence until the recent formation of IMPC (Mao, 2020; Mao and He, 2021). Such being the case, it is worth probing into different proposals of pragmatic competence and finding out the core properties of pragmatic competence for the construction of a common framework, given that a uniform concept of pragmatic competence is very essential for investigating the nature of language proper, L2 or Ln pragmatic acquisition, teaching and assessment, as well as the neurobiological and pathological manifestation of language use.

### Features of Miscellaneous Models of Pragmatic Competence

In theoretical and applied linguistics, there are roughly three pragmatic-competence-oriented research perspectives, namely, sociocultural communicative (also componential), functional, and cognitive dimensions (cf. Mao and He, 2021). Various models and theories from the three perspectives have spread over the theoretical and empirical linguistic fields, expanding into neighboring domains in cognitive science, such as the neurobiological and pathological studies of pragmatic competence (cf. Paradis, 1998; Geurts et al., 2020).

Hymes (1972), in opposition to the emphasis on abstract and decontextualized knowledge by theoretical linguists, first conceptualizes communicative competence, which involves language use by fully taking sociocultural factors into consideration. The philosophy of Hymes' practice is that sociocultural factors are what really matters in language learning. It is because children develop a theory of language use in a given context when acquiring their own grammatical competence, although they entirely experience insufficient inputs, no matter linguistic or sociocultural ones.

Heavily influenced by the notable idea of Hymes, sociolinguists and applied linguists first proceed with the investigation of what constitutes the communicative competence, that is, statically listing out specific knowledge components involved in language use. Canale and Swain (1980) and Canale (1983) specifically formulated the communicative components for the sake of language teaching, with the proposal of Canale (1983) more noticeable. The improved version of Canale (1983) consists of four components, namely, grammatical, sociolinguistic, strategic, and discourse competence. The grammatical component agrees with the classic competence, including the knowledge of lexis and rules of syntax, morphology, semantics, and phonology. The sociolinguistic competence explains why an interlocutor can interpret and produce utterances properly in a context. The strategic competence refers to verbal and non-verbal strategies used by communicators to avoid communicative breakdown, and the discourse competence enables interlocutors to adhere to the appropriate, cohesive, and coherent conversation (cf. Canale, 1983). Although pragmatic competence is not explicitly stated in their models, “pragmatics is subsumed under the discourse competence” as Ifantidou (2014: 125) predicted. This comment seemingly agrees with the late interpretation of the pragmatic competence of Canale (1988) as “illocutionary plus sociolinguistic competence.” That is to say, pragmatic competence in their system is locked down onto the level of sociocultural communication, though linguistic resources also seem indispensable.

Bachman (1990) and Bachman and Palmer (2010) furthered the componential analysis of communicative competence in terms of language assessment, formulating a well-known model—the communicative language ability (CLA)—which includes language competence, strategic competence, and psychophysiological mechanism. In comparison, it is clear that CLA remodels the proposal of Canale and presents a more practical and operable action plan that is obligatorily demanded by language assessment. In detail, pragmatic competence, singled out for the first time in the communicative framework, stands with organizational competence within language competence. Under this proposal, the language competence becomes more prominent, since its first component—pragmatic competence—is classified into illocutionary and sociolinguistic competences, as Canale proposed earlier, and the second component includes grammatical and textual competences. In this case, it is not difficult to figure out that CLA still centers on communicative issues, but absorbing certain assumptions from theoretical linguistics, namely, a parallel between grammatical and pragmatic competences as Chomsky (1977) puts forward (for detailed analysis, see Mao and He, 2021).

The communication-centered tendency is also upheld by Rose and Kasper to address pedagogical issues in interlanguage pragmatics. Rose (1999, p.171) constructed the first working concept for pragmatic competence in interlanguage pragmatics to the extent that communicators are able to use available linguistic resources to realize speech acts or doing things with words properly in social communication. Furthermore, Kasper and Rose (2002) described pragmatic competence as the ability to produce and comprehend utterances or discourse in sociocultural interactions. Notably, the pragmatic competence in interlanguage pragmatics is closely related to the interactions between linguistic resources and sociocultural contexts.

**Figure 1:**
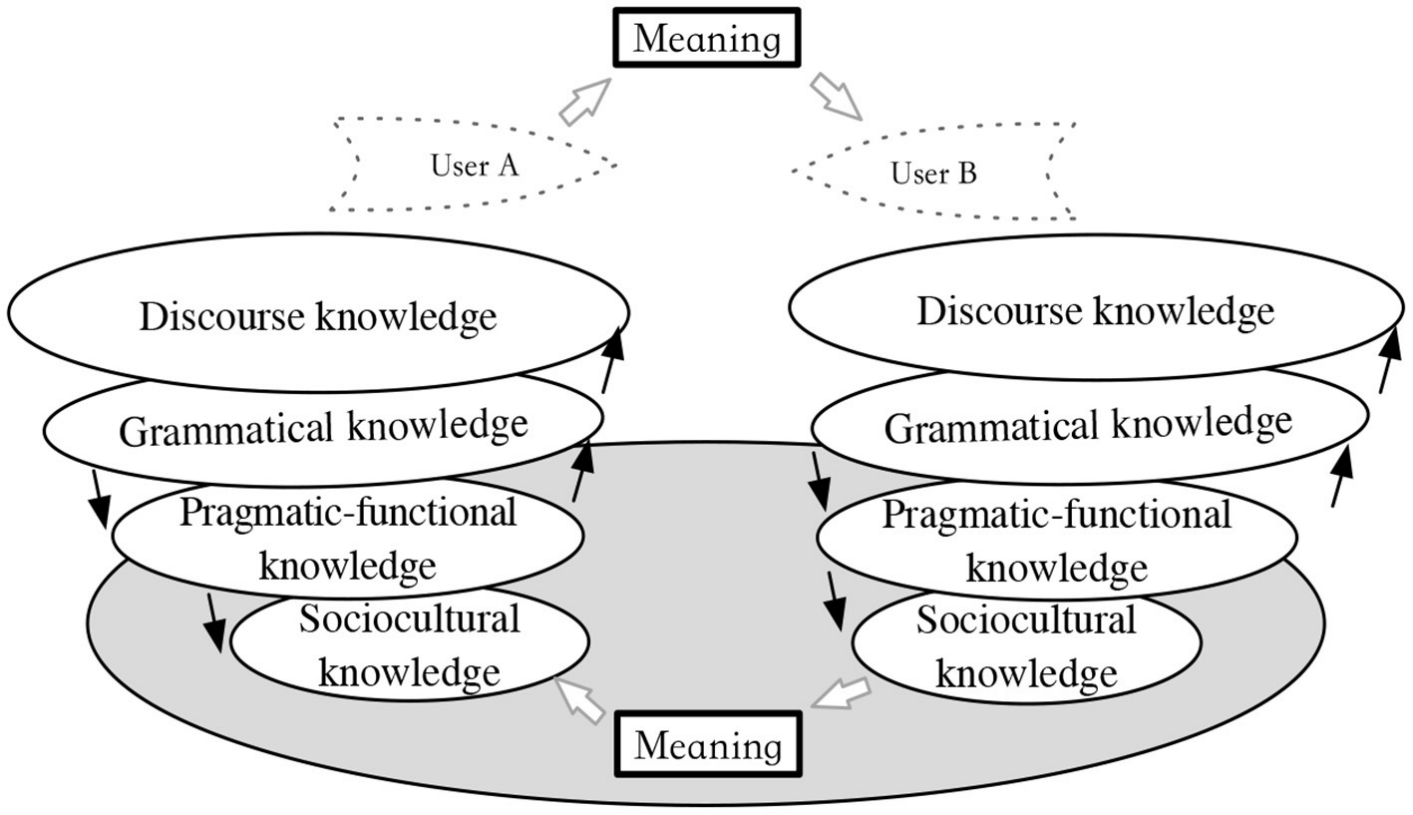
Componential and meaning-driven model (Timpe et al., 2015).

A current updated comprehensive version of the communicative competence is the componential and meaning-driven model of Timpe et al. (2015) (for a detailed review, see Timpe et al., 2015). This model novelly arranges componential knowledge in order in terms of explaining language use, with explicit emphasis on dynamic interactions between grammar and pragmatics. Undoubtedly, this is a big step forward in the reconceptualization of pragmatic competence when concentrating on dynamic interactions between relevant components. To wit, five independent but sequentially connected knowledge components push the operation of pragmatic competence during social–cultural communication. First, sociocultural knowledge (e.g., background information) functions as the basis directly related to the situational context of a communicative encounter. Second, pragmatic functional knowledge (e.g., illocutionary and sociolinguistic knowledge) connects form with function. The third level of grammatical knowledge and the fourth level of discourse knowledge draw upon Canale (1983), respectively, providing linguistic forms and methods to use them cohesively and coherently. The final dimension is the strategic knowledge that facilitates the ordered interactions between knowledge components (Figure 1, Timpe et al., 2015, p. 16).

In this way, pragmatic competence is “the dynamic and interactive negotiation of intended meaning between two or more individuals in a particular situation” (Timpe et al., 2015, p. 14). In this study, there is a very intriguing question worth considering, i.e., for what reason(s) the knowledge components are arranged in the abovementioned order during their interactions. In other words, the componential and meaning-driven model seemingly presupposes a particular theoretical background, but unrevealed in this model.

Interestingly, although functionalists firmly adhere to their philosophy of form-function mapping that exhibits how language use unfolds, they also bear the communicative end along with communicative models. For Halliday (1973), it is the function of sociocultural contexts that mediates meanings into language use. In other words, the ideational, interpersonal, and textual functional components in the semantic system set the contexts and determine the function of lexicogrammatical groups for communicative purpose. The form-function mappings realize the communicative end that pragmatic competence is supposed to reach. Similarly, van Dijk (1977) elucidates pragmatics as a theory of action that sub-serves the interactions between utterances and speech acts or social conventions, i.e., interpreting linguistic acts in sociocultural contexts.

So far, the striking characteristics of pragmatic competence models in communicative and functional perspectives are highlighted. Among all the proposals about componential knowledge concerned, two types of knowledge can be readily singled out, i.e., linguistic and sociocultural knowledge. This shared point is reminiscent of the dichotomy of pragmalinguistics and sociolinguistics of Leech (1983). The former tends to be a more linguistic end of pragmatics (i.e., linguistic resources for expressing particular illocutions); the latter refers to the more culturally specific conditions on language use. In this case, with pragmalinguistic competence, communicators can figure out which pragmatic forces certain linguistic forms can convey at an abstract thinking level, and sociopragmatic competence tells communicators whether certain speech action is appropriate in sociocultural contexts. When a speaker acquires pragmalinguistic competence, it does not necessarily mean that he/she would be a competent language user, for sociopragmatic competence tightly connects the linguistic resources with specific contextual language use. Therefore, the communicative componential models of language use underpinned by the dichotomy of Leech (1983) are seemingly in accordance with the complementary assumption of grammatical and pragmatic competences of Chomsky (1977), although the latter proposes the dichotomy with a bias to a pure linguistic end and less attention on sociocultural language use at the linguistic framework of the 1970s. In the current Minimalist Program of biolinguistic paradigm (Chomsky, 2005, 2015), the linguistic framework is reshaped to a large degree, but with the dichotomy intact of Chomsky (1977). Meanwhile, the minimalist linguistic framework regards “interfaces as the only linguistically significant levels” (Chomsky, 2000, p. 113), which has exerted heavy influence over different linguistic subfields, such as first language acquisition (cf. Guasti, 2016), second language acquisition (cf. Slabakova, 2016), and pragmatic competence reconstruction (cf. Mao, 2020) (To put aside the topic for the moment and discuss it later).

Under the third perspective (cognitive stance), cognitive pragmatists spare no efforts to dig into the basic properties and operative mechanisms of pragmatic competence. For instance, Ifantidou (2014), based on relevance-theoretic assumptions (Sperber and Wilson, 1995), considered pragmatic competence as the underlying mechanism for both abstract thinking and sociocultural communication. In detail, pragmatic competence comprises three-level interactive ability: (1) recognizing relevant linguistic indexes (Linguistic awareness); (2) picking up relevant pragmatic effects (Pragmatic awareness); and (3) clarifying the link between lexical indexes and pragmatic effects retrieved (Metapragmatic awareness) (Ifantidou, 2014, p. 130). Accordingly, the linguistic awareness aids interlocutors to find out linguistic cues of structures, and interlocutors with pragmatic awareness associate linguistic forms (recognized by the cues) with pragmatic effects (pragmalinguistic awareness). Based on the stable connection, metapragmatic awareness facilitates interlocutors to know and/or use proper linguistic forms in proper contexts (sociolinguistic awareness). Evidently, the tripartite cognitive model boosts a dynamic operation of pragmatic competence on the basis of interactions between different modules of knowledge (e.g., syntax and semantics). Totally diverged from the abovementioned componential models, this proposal introduces a promising cognitive trend in investigating pragmatic competence, which definitely agrees with the current advancement in cognitive science. However, one point is worth mentioning, although this proposal subsumes both communicative and thinking activities, pragmatic competence contains grammatical competence rather than that forms a complementarity with it, since “metapragmatic awareness requires linguistic awareness to identify linguistically encoded phenomenon^1^” (Ifantidou, 2014, p. 149).

Along this cognitive trend, Kecskés explicitly takes pragmatic competence rather than communicative competence as the core because of “the difficulty to draw a line between pragmatic competence and intercultural communicative competence” (Kecskés, 2013, p. 61). Due to this particular viewpoint, there is only one uniform pragmatic system in interlanguage and intercultural pragmatic development (Kecskés, 2015, p. 420), and bilinguals just modify the pragmatic competence of their L1s to desirable L2 and Ln pragmatic competence with access to novel sociocultural norms and conventions of new languages. During the pragmatic acquisition, it is primarily the conceptual change rather than the ancillary linguistic socialization that forges a transition from L1 pragmatic competence to those of L2 and Ln. As is clear, this proposal values the dynamic cognitive pragmatic development facilitated by the sociocultural conventions. With the shared cognitive properties stabilized, the unfolding of pragmatic competence naturally follows thereby.

Undoubtedly, given that language development is primarily a matter of cognitive development rather than piecemeal sociocultural accumulation, the cognitive trend in the investigation of pragmatic competence is more attractive and convincing. To admit, the communicative and functionalist models closely reveal the sociocultural aspect of pragmatic competence, disclosing how the sociocultural factors advance language use. However, what roles cognitive agents play in sociocultural communication is not clearly exhibited. To put it another way, the majority of communicative models or the relevant ones merely expound what componential knowledge involves in language use during sociocultural communication (e.g., statically listing relevant knowledge components), less caring about how communicators as active cognitive agents dynamically make use of knowledge components to achieve their communicative purposes. Of course, this move might not be seriously blamed, given its sociocultural communicative origin.

Chomsky (2011, p.266) commented on the definition of “communication,” namely, “statistically speaking, language use is overwhelmingly internal—speaking to oneself; If one chooses to call this ‘communication' …then imagined social context is relevant.” That is to say, in communication, language use could denote two basic levels. One is to realize explicit sociocultural communication, and the other covers abstract thinking activities, such as thinking by language means about communicative or non-communicative issues in imagined or even authentic contexts (cf. Mao, 2020). Under the latter cases, the communicators definitely use language to carry out cognitive thinking to fulfill linguistic and communicative purposes. These speculations agree with the explanation of language proper as “primarily designed as an instrument for construction and interpretation of thought” (Berwick and Chomsky, 2016, p. 102), or “fundamentally a system of audible signs for thought” (Whitney, 1908, p. 3). If this thought is on the right track, the cognitive trend in the investigation of pragmatic competence should be treasured, with two basic properties of language realized during the unrolling of pragmatic competence. In other words, a reconceptualization of pragmatic competence should serve to the implementation of basic properties of language—primarily for thought and then for communication.

Furthermore, in the tripartite cognitive model of pragmatic competence, regardless of pragmatic competence subsuming grammatical competence, or vice versa, the pair forms their independent modular systems, namely, they might form a special complementary relation as mentioned in the study by Chomsky (1977). In the proposal of Kecskés (2013, 2015), the complementary dichotomy of Chomsky is seemingly obeyed because of the uniformity of pragmatic competence systems in different languages. This complementary tendency can also be checked in the communicative models of pragmatic competence (cf. Mao and He, 2021). If the verification of complementarity between grammatical and pragmatic competences is on the right way, this valuable practical guiding principle stands firm for the reconstruction of the operative mechanism of pragmatic competence, no matter in what linguistic schools/subfields or in various neighboring fields (for the same view, see Mao and He, 2021).

### Essence of IMPC

The twenty-first century is the century of brain science. In this special time, modern neuroscience has entered the era of intellectualization and precision. The accurate functional and anatomical analyses of the neural representations of linguistic computations can testify whether “the architectural design of language faculty” (Chomsky, 2005) is feasible. For example, Friederici (2017) corroborates that Brodmann areas 44 and 45 are fully engaged in pure syntactic and semantic computations, respectively. That is to say, at least, the individual functional linguistic modules work together to realize linguistic computations. This tendency fully matches the interface-centered linguistic inquiry in the current Minimalist Program of biolinguistic paradigm (cf. Chomsky, 2000; Mao, 2020), solidifying the foundation for the earnest exploration of pragmatic competence mechanism underlying dynamic language use.

#### Internal Pragmatic Competence

**Figure 2:**
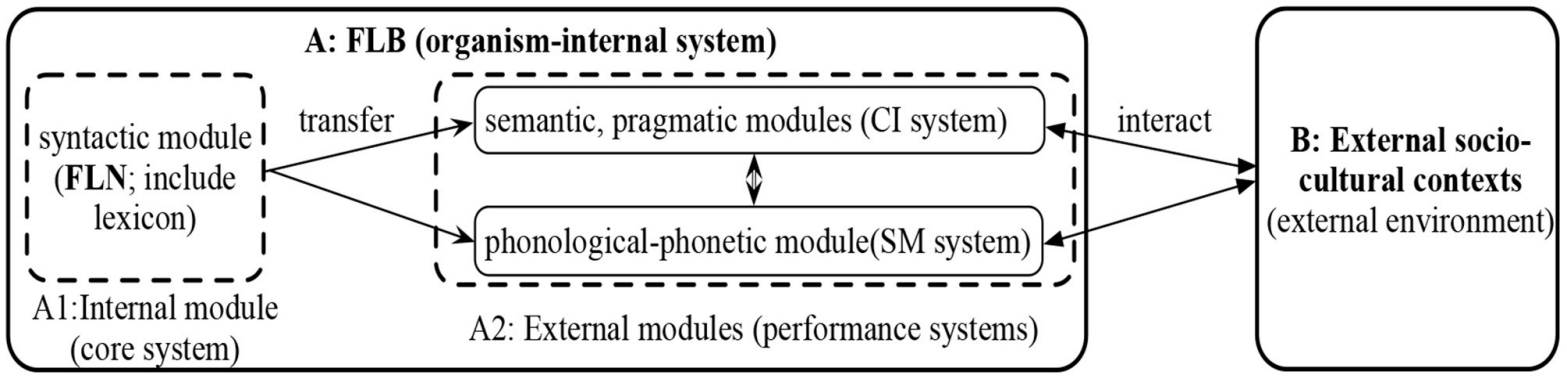
Intreractions in FLB and sociocultural contexts (Mao, 2020).

To address the Descartes' Problem listed out by Chomsky (2007, p. 14–15) for the biolinguistic research, i.e., explaining “how I-language is put to use” (“I” stands for internal, individual, and intentional), Mao (2020) and Mao and He (2021) recently proposed IMPC in the current minimalist framework of biolinguistic paradigm. The basic motivation of IMPC is to check how I-language, as an internal biological object with the genetically determined generative procedure and an internalized linguistic system or internalized linguistic knowledge (cf. Chomsky, 2015), can be exploited to reach various kinds of human ends. Under the assumptions of IMPC, pragmatic competence underpins the strong and weak versions of linguistic performance, namely, facilitating both the actual use of language and the mental processing of linguistic representations in use (Mao and He, 2021). Hence, the dynamic operation involves interactions between all linguistic subsystems (e.g., syntax and semantics) of the language faculty in the broad sense (FLB, Hauser et al., 2002) and their interactions with other cognitive systems or contexts outside FLB, as shown in Figure 2 (Mao, 2020).

The designed connections among modular linguistic subsystems originate from the assumptions of FLB and the language faculty in the narrow sense (FLN, Hauser et al., 2002). To wit, FLN (the syntactic module) functions as the engine to generate linguistic representations to satisfy the legibility conditions imposed by two performance systems in FLB, namely, the conceptual-intentional (CI) system and the sensory-motor (SM) system. In other words, what the narrow syntax sub-module generates *via* recursive Merge should be usable by semantic and pragmatic sub-modules in the CI system and phonological-phonetic sub-module in the SM system. On the contrary, given the complementarity between grammatical and pragmatic competences, if cognitive agents, in CI and SM systems, can judge the usability of linguistic representations transferred from the syntax sub-module, it definitely means that they are equipped with tacit internal pragmatic competence (IPC); that is to say, they have an idea of how to use linguistic representations grammatically and properly in their minds. The modular-interaction-based pragmatic competence thus subserves the dynamic use of the internal language at the abstract thinking level. To cite one example to demonstrate the operative mechanism of IPC running along this route map. (1) is selected from a short essay by an L2 Chinese learner about a 联欢会 (*lián huān huì* “gathering”) on Christmas Eve.

(1) ^*^我觉得跟别人联欢会有更丰富的感觉

Wo juédé gēn biérén liánhuānhuì you gèng fēngfùde gǎnjué

I think with others gathering have more rich feeling

“I think there is a rich feeling to have a gathering with others.” Along the operative procedure in Figure 2, the L2 Chinese learner generates the syntactic representation of (1) *via* Merge and then transfers it to the semantic module for interpretation. However, 跟 (*gēn* “with”) as a proposition here cannot assign two thematic roles, but only one (i.e., theme) to the closest argument, namely, (跟, P [theme]). 联欢会 (*lián huān huì* “gathering”) is thus semantically deviant. In this case, the event denoted by (1) cannot be interpreted in the semantic sub-module, except that one thematic role assigner is added before *lián huān huì* “gathering” in the syntactic submodule, such as 开联欢会 (*kāi lián huān huì* “have a gathering”). If the L2 Chinese learner can make use of the uninterpretability of (1) to recognize the ungrammaticality of (1) at the abstract thinking level, he/she is definitely endowed with IPC. If not, he/she cannot tacitly manipulate the interactive mappings among different sub-modules to use language correctly, thus, an imperfect IPC.

Such being the case, IPC is realized by the interactions between internal syntactic sub-module with external but organism-internal sub-modules, such as semantics, pragmatics, and phonology-phonetics (i.e., A1 → A2 in Figure 2), and between the external but organism-internal sub-modules within A2, or all the organism-internal sub-modules interacting with outer sociocultural situations at the abstract thinking level in authentic or imagined contexts: (A1 → A2)↔B. Given that the modular interactions at the abstract thinking level define the operation of IPC, it is not difficult to note that IPC truly reveals the primary property of language and language use, namely, prioritizing the abstract thought in essence. In this case, it is not surprised for communicators to adopt IPC to abstractly think about communicative issues in real or imagined contexts.

### Pragmatic Competence for External Communication

As is well-known, language is a vital tool in daily communication, enabling common people to perform various speech acts and convey their intentions in sociocultural communication. In this case, the language system not only fulfills the duty to realize thoughts as IPC demonstrates but also facilitates daily sociocultural communication.

Ideally, competent interlocutors know how to utilize pure linguistic knowledge and socio-pragmatic knowledge (as proposed in the abovementioned communicative, functional, and cognitive models) to communicate with each other. Under IMPC, it means they can properly use the computational results from all the organism-internal sub-modules in sociocultural contexts, namely, (A1 → A2) ↔B. More technically, the smooth unrolling of pragmatic processing is underpinned by the same pragmatic competence mechanism but targeting at sociocultural communication; thus, a pragmatic competence for external communication (PCEC) can be recognized (Mao, 2020).

During the operation of PCEC, the third-factor principle, i.e., the relevance, between the linguistic (syntactic, semantic, and phonological-phonetic) representations and socio-pragmatic knowledge maintains the felicitous mappings of linguistic computational results into corresponding contexts. That is to say, the relevance helps interlocutors to retrieve relevant contextually proper pragmatic knowledge from background or encyclopedia knowledge and put it into the pragmatic sub-module to reason out the pragmatic meaning. For example, A and B meet at a dinner party held by Mrs. White,

(2) A: Mr. White blamed his wife right now, right?
  B: The food is very delicious, isn't it?

Evidently, B “fouled up” the conversational turn by responding uncooperatively. However, considering polite communication, B properly behaved to avoid the embarrassing topic, namely, an excellent PCEC dispels the embarrassment to talk about the privacy of someone else. In detail, after sequential syntactic and semantic computations, B reached the plain meaning of A: Mr. White hurt his wife or treated his wife badly. Furthermore, against the context, B retrieved the relevant pragmatic knowledge from background information, i.e., it is impolite to comment on someone openly and better to change the topic. The pragmatic processing, based on serial interactions between different subsystems of the language faculty and the relevance between linguistic and pragmatic knowledge, explicitly manifests the unfolding of PCEC. As evidenced, the duty of PCEC to propel sociocultural communication by language means is achieved during its operation.

To summarize, IMPC relying on modular interactions and beyond faithfully realizes the dual nature of language or language use, i.e., first functioning as a species-specific vehicle of thought and then as a tool of communication (i.e., two sides of one coin). Meanwhile, during the execution of IMPC, it not only involves pure linguistic knowledge and socio-pragmatic/pragmalinguistic knowledge as other models proposed but also assumes a complementarity between grammatical and pragmatic competences. Based on the latter, the interactions between the pair and beyond exhibit how the internal language is used.

### Common Core of Various Models of Pragmatic Competence

The study of pragmatic competence is one of the strategic objectives to enable whatever linguistic explorations to hit the ground to serve linguistic ends and real social needs (another fascinating move is the neurobiological investigation of language proper and its acquisition and use). Diachronically, various linguistic schools or researchers have proposed different models or projects to delineate how language is used to realize sociocultural communication and abstract thinking activities (including silent communication). Undoubtedly, the existing investigations as explicated earlier, no matter they contradict with each other or bias their choices to their own theoretical interests, are definitely conducive to disclose the universal properties of pragmatic competence and construct a more reasonable operative mechanism of pragmatic competence. In this case, the common core of diverse constructs, mechanisms, and models/theories of pragmatic competence is very precious to us.

The most impressive property revealed by the existing proposals of pragmatic competence is that the models realize either authentic sociocultural communication or pure thought, or both functions. This is a primary desideratum for almost all linguists because the dual nature of language use, namely, thinking and communication, is completely unveiled. In detail, an ideal model of pragmatic competence should cover all aspects of language use, namely, the formation of internal linguistic system to execute thinking activities and the externalization of linguistic computational results from the internal linguistic system to realize sociocultural communication. Therefore, the formation of internal language for thought and the externalization of linguistic representations for communication are like “two sides of one coin.” Only in this way can the proposal comprehensively uncover the two-sides-of-one-coin nature of language use (or language proper). For instance, the communicative componential models are strongly biased to one side of language use, i.e., sociocultural communication, so do functionalists, similar to van Dijk (1977), and other models based on communicative competence (cf. Timpe et al., 2015; Mao and He, 2021). As a step forward, cognitive models or proposals manage to cover both sides of language use, such as the relevance-theoretic tripartite cognitive model (Ifantidou, 2014). Finally, IMPC, rooted in the Minimalist Program of biolinguistics, stands with the practice of tripartite cognitive model, assuming a unanimous two-part pragmatic competence. Specifically, IPC sub-serves the abstract thinking activities (including silent communication) and PCEC for the authentic contextual communication. The operative mechanism of IMPC thus realizes the two sides of language and language use. In this manner, a panoramic perspective comes into existence, which fosters cognitive scientists more efficiently to deconstruct the core properties of pragmatic competence or language *per se*.

The bonus to construct a pragmatic competence mechanism such as the two-part IMPC to unwrap the two-sides-of-one-coin nature of language use lies in the formation of a uniform pragmatic competence mechanism in various domains, without differentiation of the first from the second language and pragmatic competence from communicative competence. It is because the same core mechanism of pragmatic competence readily brings the relevant theoretical and empirical investigations together, and the variation only consists of the developmental patterns in the first and second languages (Kecskés, 2013; Mao and He, 2021). In this case, the unique pragmatic competence mechanism, as proposed by IMPC and others, could guarantee pragmatic competence as an integrated concept that implements both thinking and sociocultural communication. This move evidently makes the investigation of the nature of pragmatic competence, its operation, and acquisition more attainable and easily intercommunicated among researchers. More importantly, it enables the exploration of the design of language architecture to approach much closer to the truth.

Furthermore, Timpe et al. (2015, p. 9) cautioned that how different components of communicative competence dynamically interact remains open to speculation while it accepts the interconnectedness of pragmatic, grammatical, and sociocultural components at the communicative levels. In other words, to accurately expound the dynamic character of language use is a very crucial issue for investigating the nature of pragmatic competence and its operative mechanism. For communicative and functionalist proposals, knowledge representations rather than derivational computations come into focus. This is seemingly delimited by their theoretical frameworks, namely, mostly adopting a descriptive and prescriptive but not explanative methodology to analyze the linguistic phenomena. Such being the case, it is not a surprise to find out that their constructs, models, or theories of pragmatic competence are built on static knowledge components instead of dynamic interactions between different pure linguistic and pragmatic knowledge or beyond.

In this aspect, cognitive models of pragmatic competence carry researchers a step forward. The tripartite cognitive model acknowledges the form-function mapping emphasized by functionalists, subsuming a three-level interactive ability to explain how language use dynamically proceeds with the aid of relevant connections among three linguistic or metalinguistic levels. Along this line, IMPC, based on a well-proved derivational approach of the minimalist practice, dynamically redesigns how pragmatic competence unfolds, namely, attributing the operation of pragmatic competence to the dynamic serial interactions between several organism-internal sub-modules and their interaction with authentic sociocultural contexts. The relevance, as the third-factor principle, drives the non-linguistic knowledge in corresponding sub-modules to realize the dual nature of language use, i.e., thinking and communication. In doing so, the dynamic initiative in the tripartite cognitive model and IMPC seemingly file a positive solution to the concern of Timpe et al. (2015).

If the satisfaction of dynamic exploration of pragmatic competence and its mechanism is on the right track, this requirement presupposes a tight relation between grammatical and pragmatic competence, as disclosed in the three perspectives and the bio-linguistic standpoint (i.e., IMPC). The reason is that it is impossible for pragmatic competence to implement dynamic interactions without real linguistic entities. Most importantly, as the tripartite cognitive model and the bio-linguistic model recognize that grammatical competence lays the foundation for the unrolling of pragmatic competence, the pragmatic competence bridges the grammatical competence with contextual realization, that is, to facilitate grammatical competence to meet the legibility conditions imposed by linguistic performance systems (see “The core of pragmatic competence” section). As a result, the complementarity between grammatical competence and pragmatic competence, drafted by Chomsky (1977), plays an essential role in investigating pragmatic competence (for a detailed verification, see Mao, 2020), in particular, in designing the operative mechanism, which points out the departure for further studying pragmatic competence.

## Route Map for Further Exploration of L2/Ln Pragmatic Competence

If the abovementioned recapitulation of core properties of pragmatic competence or its mechanism is reasonable, it is possible to draft a course of action to investigate the pragmatic competence in the new century, which will solidify the foundation for the clarification of its definition, mechanism, acquisition, and assessment.

### From Sociocultural Exploration to Examination of Abstract Thinking

Undoubtedly, pragmatic competence underpins the daily communication of the people. Communicative competence models have taken this fact as a starting point to scrutinize the constituents, teaching, and assessment of communicative and/or pragmatic competence. The empirical research along this schedule informs linguistic circle or its neighboring fields a lot, because models and theories serving the explanation of sociocultural communication reflect one side of language or language use, though a clearly defined operative mechanism is not the focus for most researchers in this perspective.

Be that as it may, language is an indispensable vehicle to carry out pure abstract thinking activities as demonstrated by Berwick and Chomsky (2016). Along this path, the investigation of how I-language is put to use inevitably necessitates constructing of an operable mechanism that facilitates how abstract thinking activities unfold by linguistic means. If this assumption sounds reasonable, how human thought unfolds needs further specification. As is evidenced, the static listing of knowledge components is not the best way to probe into the nature of language use. It is because language use is, in nature, a dynamic rather than static process, and the static knowledge components cannot display how the human brain, i.e., the black box, operates in language use. In this case, learning from the current achievements about modular interactions in brain science appears to be a promising move for the investigation of the pragmatic competence mechanism underlying language use.

More specifically, relying on the synchronization between anatomical and functional modularity in the human brain (cf. Grodzinsky and Santi, 2008; Friederici, 2017) and the modular interactions, it is possible to integrate different kinds of static knowledge components into dynamic linguistic processing in the human brain. The interactions between modules of syntax, semantics, and phonology-phonetics exhibit the dynamic linguistic processing in language use. The IMPC is such a model that tentatively implements this route map to make known pragmatic competence mechanism and the dynamic nature of language use. For instance, under IMPC, on the one hand, the modular interactions and their interactions with authentic situations manifest how pragmatic competence operates during language use (or how I-language is put to use) (see “The core of pragmatic competence” section), realizing pure thought by IPC and sociocultural communication through PCEC. On the other hand, CI interfaces (or logical form in traditional terms) generated by the narrow syntax appear as the crucial means of thought after being enriched semantically and pragmatically into complete propositions with the aid of pragmatic competence (cf. Mao, 2020). That is, based on IPC, modular interactions between organism-internal sub-modules build up a language of thought for thinking about the world and other issues. However, whether IMPC is feasible or other models have more potential in pursuing this route map needs to be verified in near future.

Anyhow, if the speculations appear plausible, it would be better for the investigation of pragmatic competence to value both sides—thought and communication—and to assume the same pragmatic competence system or mechanism to fulfill two duties, namely, not only concentrating on sociocultural communication but also prioritizing thinking activities as the basic tenet. In other words, the pragmatic competence mechanism should be designed to exercise two functions that correspond to the nature of “two sides of one coin” of language and language use.

### From Typical Investigation to the Examination of Atypical Development

In general, the majority of L2 or Ln pragmatic competence studies are biased to how sociocultural factors influence and facilitate L2 or Ln typical learners to use language properly and acquire pragmatic competence. Although the definition and mechanism of pragmatic competence are far from unanimously accepted, the relevant research on normal pragmatic competence development has accumulated a wealth of empirical and theoretical bases for further investigation of pragmatic competence.

With the rapid development of modern brain science and neuroscience, the neurobiological exploration of the properties of language and its acquisition and use has provided linguists with valuable evidence to improve the design of language architecture and theories about linguistic computations, acquisition, and use. Among the abundant neurobiological research on language, the pathological and bio-linguistic investigation of impressive symptoms such as autism spectrum disorder (ASD), Williams syndrome (WS), and specific language impairment (SLI) has revealed a lot about language itself and its acquisition and use (cf. Mao et al., 2020). Especially, autism, featured by lingering socio-pragmatic deficits and difficulties among various language learners and users (American Psychiatric Association., 2013), functions as a solid pathological evidence to expose the nature of pragmatic competence.

According to the official data, there is a steady increase in diagnosis rates of autism from 1 in 150 children in the United States in 2000 to 1 in 59 in 2016 (Morsanyi and Byrne, 2020). Also, Kim et al. (2014, p.268) reported that 30% of children with ASD never achieve verbal communication. In this case, the exploration of the unique linguistic profile of people with ASD could inform linguists and speech therapists of the nature of language and its use. Kissine (2020) pointed out that most autistic people turn out to be egocentric language learners and users. This particular profile might or might not originate from the difficulties in mind reading, which are inherent in the autism diagnosis (cf. Geurts et al., 2020). For the moment, putting aside the possible hurdle in direct communication and focusing on the egocentric linguistic computations of autistic individuals, people may wonder what the potential mechanism(s) autistic individuals adopt to conduct thinking and silent communication with themselves. Can IMPC function as the possible mechanism explaining the unique linguistic profile of autistic individuals? In detail, under IMPC, whether autistic individuals resort to IPC and PCEC for egocentric thinking and (silent) sociocultural communication, respectively, is not clear. If IMPC is right in this aspect, could it uncover the core properties of language use as a whole? If not, are there extra methods by other models of pragmatic competence or language use that are able to clarify the unique linguistic profile of autistics or the common core of L2 or Ln pragmatic competence? Either way, a better understanding of the thinking pattern(s) of people with ASD is helpful to those who are related to autistic individuals, such as clinicians and their families, to aid autistic individuals to improve language acquisition and use.

Kissine (2020) also pointed out that unveiling the basic properties of language use of autistic people is seemingly beneficial to understand the existing theoretical gap between non-intersubjective and intersubjective language use and acquisition, i.e., exposing the discrepancies between nativism and constructionism. In this case, the pathological and the biolinguistic investigation of impaired language use would usher in a new perspective to catalyze the exploration of the nature of language use, its mechanism, acquisition, and assessment, as well as language proper in-depth.

## Conclusion

With the advent of the new century, Chomsky (2007) made a plan for linguistic inquiry, among which the Descartes' Problem stands out to expound how the internal language enters into various kinds of uses and aims to probe into the topics as neo-Gricean pragmatics does, such as acts of referring to the world and interchange with others. Evidently, this is the logical end of theoretical and empirical research in generative linguistic inquiry or any other linguistic perspectives. Undoubtedly, this proposal enables the investigation of pragmatic competence to be readily integrated into the minimalist inquiry of the biolinguistic paradigm. The other way around, the theoretical and empirical lessons and achievements obtained in the minimalist inquiry have solidified the foundation to reinvestigate the core properties and operative mechanism of pragmatic competence, as well as its acquisition and assessment.

If this task fits well with the research interests of other linguistic perspectives and models, the common core of pragmatic competence elaborated here accounts for a significant starting point for the next move in elucidating pragmatic competence in a panoramic way. At least, the common core includes (but not limited to) the grammatical and pragmatic knowledge components clarified by the communicative componential models and the meaning-driven models of Timpe et al., the dynamic form-function mapping delineated by functionalists, the tripartite cognitive models and IMPC, as well as the integration of thought and communication and assumption of one uniform pragmatic mechanism in IMPC. All these findings, together with the lessons detailed in this study, will further propel an intensive study of pragmatic competence in the new century.

Besides, the pathological and neurobiological investigation of pragmatic impairment is another genuine growth point to reshape the models or theories of pragmatic competence. More promisingly, on the one hand, the explication of pathological symptoms and the neurobiological mechanism of language use absolutely makes research of pragmatic competence meet the realistic requirements of human society; on the other hand, this type of examination definitely extends valuable feedback to the improvement of linguistic theories, including various proposals for grammatical and pragmatic competences. Bearing all the above in mind, the investigation of the cognitive scientist about pragmatic competence, regardless of L2 or Ln, would be endowed with a new opportunity to move forward in the coming years.

## Data Availability Statement

The original contributions generated for the study are included in the article/supplementary material, further inquiries can be directed to the corresponding authors.

## Author Contributions

The author confirms being the sole contributor of this work and has approved it for publication.

## Conflict of Interest

The author declares that the research was conducted in the absence of any commercial or financial relationships that could be construed as a potential conflict of interest.

## Publisher's Note

All claims expressed in this article are solely those of the authors and do not necessarily represent those of their affiliated organizations, or those of the publisher, the editors and the reviewers. Any product that may be evaluated in this article, or claim that may be made by its manufacturer, is not guaranteed or endorsed by the publisher.

## Footnotes

^1^The reason is that grammatical or linguistic awareness is important to help communicators recognize or identify the linguistic indexes of utterances for subsequent pragmatic reasoning.
